# Flexible, diamond-based microelectrodes fabricated using the diamond growth side for neural sensing

**DOI:** 10.1038/s41378-020-0155-1

**Published:** 2020-07-13

**Authors:** Bin Fan, Cory A. Rusinek, Cort H. Thompson, Monica Setien, Yue Guo, Robert Rechenberg, Yan Gong, Arthur J. Weber, Michael F. Becker, Erin Purcell, Wen Li

**Affiliations:** 1 Department of Electrical and Computer Engineering, Michigan State University, East Lansing, MI USA; 2 Fraunhofer USA Center for Coatings and Diamond Technologies, East Lansing, MI USA; 3 Department of Biomedical Engineering, Michigan State University, East Lansing, MI USA; 4 Department of Physiology, Michigan State University, East Lansing, MI USA

**Keywords:** Electrical and electronic engineering, Chemistry

## Abstract

Diamond possesses many favorable properties for biochemical sensors, including biocompatibility, chemical inertness, resistance to biofouling, an extremely wide potential window, and low double-layer capacitance. The hardness of diamond, however, has hindered its applications in neural implants due to the mechanical property mismatch between diamond and soft nervous tissues. Here, we present a flexible, diamond-based microelectrode probe consisting of multichannel boron-doped polycrystalline diamond (BDD) microelectrodes on a soft Parylene C substrate. We developed and optimized a wafer-scale fabrication approach that allows the use of the growth side of the BDD thin film as the sensing surface. Compared to the nucleation surface, the BDD growth side exhibited a rougher morphology, a higher *sp*^3^ content, a wider water potential window, and a lower background current. The dopamine (DA) sensing capability of the BDD growth surface electrodes was validated in a 1.0 mM DA solution, which shows better sensitivity and stability than the BDD nucleation surface electrodes. The results of these comparative studies suggest that using the BDD growth surface for making implantable microelectrodes has significant advantages in terms of the sensitivity, selectivity, and stability of a neural implant. Furthermore, we validated the functionality of the BDD growth side electrodes for neural recordings both in vitro and in vivo. The biocompatibility of the microcrystalline diamond film was also assessed in vitro using rat cortical neuron cultures.

## Biosensors: Flexible diamond-based microelectrodes for neural sensing

A flexible, sensitive, stable neural probe consisting of boron-doped polycrystalline diamond (BDD) microelectrodes has been developed, whereby the BDD growth surface is used as the electrode site for neurophysiological and neurochemical sensing. Among carbon materials, BDD has found widespread use in neurotransmitter detection, but the hardness of diamond has impeded application in neural implants. A team headed by Wen Li at Michigan State University, USA, succeeded in developing a process that allows the growth side of the BDD thin film to be used as the sensing surface: they transferred the BDD patterns from a solid silicon substrate onto a flexible polymer substrate. The authors validated their BDD microelectrode technology for neural recordings both in vitro and in vivo, and they believe it has excellent potential for research into various brain disorders, such as Parkinson’s disease.

## Graphical abstract

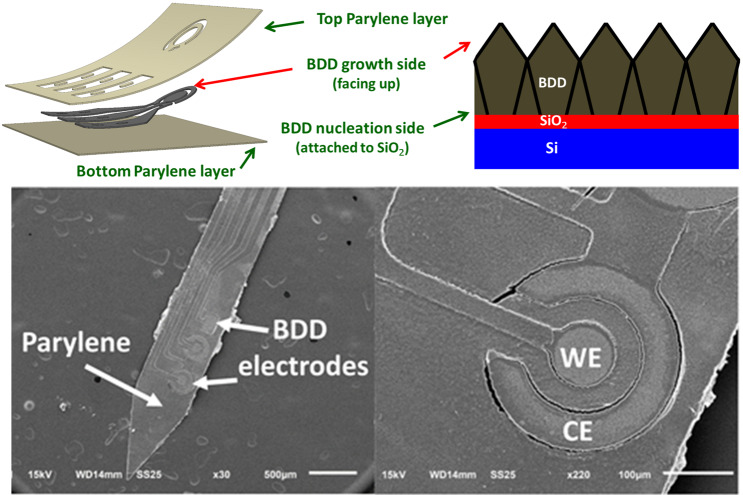

## Introduction

Nervous systems consist of large networks of neurons that communicate with each other both electrically and chemically. Rapid, transient communication in neural circuits primarily occurs via electrical impulses (action potentials) along the axon. Neuron-to-neuron communication occurs chemically through the secretion of chemical messengers, known as neurotransmitters that bind to specific receptors at synapses^1^. The transduction of neurotransmitters among the body’s glands, organs, and muscles regulates and influences complex brain functions and behaviors such as emotion^2^, pain response^3^, and mental disorders^4^. Numerous studies have shown that dysregulated or imbalanced levels of neurotransmitters in the brain, such as dopamine (DA), are associated with many neurological disorders and health conditions, such as Parkinson’s disease^5^, schizophrenia^6^, and epilepsy^7^. The ability to characterize the real-time DA dynamic release and uptake will contribute to a complete understanding of the mechanism behind these diseases and provide effective therapeutics.

Over the past few decades, tremendous efforts have been made to develop miniaturized electrode sensors based on micro/nanofabrication technologies for multimodal neurotransmitter sensing and neurophysiology recording with improved spatiotemporal resolution. For neurophysiology recording, while extremely popular in their use, metal microwires and silicon microelectrodes suffer from signal instability and degradation after chronic implantation due to inflammatory responses and fibrous encapsulation evoked by electrode insertion and the subsequent relative motion^8,9^. Increasing evidence suggests that the dimension and stiffness of implanted electrodes contribute greatly to tissue damage and fibrosis in chronic implantation^10^. As such, polymer-based electrodes have been explored extensively as an alternative to reduce the mechanical mismatch of the devices with the surrounding tissues^11^. However, the fabrication of polymer-based electrodes with small dimensions and good durability is challenging due to physical limitations, non-hermetic packaging, and tradeoffs between size and mechanical strength. While recently reported carbon fiber microelectrodes (CFMEs) have a small cross-sectional footprint (8–9 µm diameter) and a low stiffness that promise to reduce chronic tissue damage and neuron death upon implantation^12–14^, the fabrication of CFME arrays requires a laborious and time-consuming alignment and the assembly of individual fibers onto prepatterned circuit board substrates, which may not be practical for building high-channel-count arrays. For neurotransmitter sensing, the traditional microdialysis technique^15–19^ allows the detection of chemicals at extremely low concentrations but has a limited temporal resolution on the minute scale. Electrochemical approaches using microelectrodes, on the other hand, can detect faster events and therefore are more suitable for real-time DA monitoring^20^. However, detecting DA electrochemically in living organisms is extremely challenging because other chemical species that coexist with DA, such as ascorbic acid (AA), are 100–1000 times more concentrated than DA^20^. In particular, AA has been found to have an oxidation potential very close to that of DA, resulting in a significant interference^21^.

Ideally, the simultaneous probing of neurophysiology and neurotransmitter signals requires a sophisticated, implantable device that is highly sensitive and selective, miniaturized, fast, biocompatible, and long-term stable^22^. At present, most of the implantable sensors are constructed in an electrode configuration, mainly based on carbon materials, including glassy carbon^23^, carbon nanotubes^24–27^, carbon fibers^28^, and micro/nanocrystalline diamonds^29–32^. Among different carbon materials, boron-doped polycrystalline diamond (BDD) has stood out and has found widespread use in neurotransmitter detection, especially for DA^21,33–35^. Diamond is inherently an insulator, but when doped with boron, the material possesses semimetal electronic properties, making it useful for electrochemical measurements. During film growth, the boron impurity atoms substitute for some of the carbon atoms, resulting in a rough, polycrystalline morphology with grain boundaries at the film surface and a small-volume fraction of non-diamond carbon (*sp*^2^) impurity. Consequently, the electrical conductivity and electrochemical properties of the film surface and the bulk are influenced by the boron doping level, the grain boundaries, and the impurities^36^. Common BDD properties favorable to electrochemical sensing include a wide potential window, a low double-layer capacitance, excellent selectivity and sensitivity without conventional pretreatment, weak molecular adsorption, biocompatibility, and chemical inertness^37,38^. Despite the many benefits of BDD, the mechanical property mismatch between BDD (Young’s module of ~10^3^ GPa)^39^ and soft tissues (~10^3^–10^5^ Pa)^40^ is a major obstacle that impedes the development of BDD into fully implantable electrochemical devices. Many efforts have been devoted to developing various fabrication methods to make diamond-polymer structures with reduced stiffness while maintaining the biocompatibility and inertness of the overall devices. For example, Hess et al.^40^ reported a method of transferring small BDD patterns, such as contact pads and electrodes, from a silicon substrate onto a polynorbornene-based polymer (PNB) film by removing a SiO_2_ sacrificial layer between the BDD patterns and the silicon substrate. Later, Bergonzo et al.^41^ used a similar fabrication process to transfer a diamond microelectrode array onto a flexible polyimide substrate for in vivo retinal prostheses. Previously, we also reported a method for patterning and transferring large-scale BDD structures from solid substrates onto Parylene C thin films. The transferred flexible BDD electrodes have been proven effective for detecting various concentrations of DA with a limit of detection (LOD) of 0.5 µM^42^.

However, the above transfer methods allow exposing only the BDD nucleation surface as the sensing sites, keeping the growth surface encapsulated by the polymer film. The nucleation surface is the interface between the BDD film and the substrate where diamond deposition is started. The nucleation surface has small diamond grain sizes and a high density of grain boundaries with a large number of impurities, such as *sp*^2^ carbon, within the boundaries. These non-diamond impurities can significantly affect the electrochemical properties of BDD electrodes. As the film thickness increases, the diamond grain size increases, resulting in more *sp*^3^ carbon atoms and less *sp*^2^ impurities. Qi et al.^43^ reported the effects of the BDD film thickness on the electrochemical properties associated with an *sp*^2^ impurity, including the electron transfer kinetics, the faradaic current of DA, and the oxidation potential separation between DA and AA. This study has shown that 5.5-µm-thick BDD has much less *sp*^2^ impurities than 0.5-µm-thick BDD, leading to a significantly faster electron transfer process in the [Fe_3_(CN)_6_]^3−/4−^ redox system, a higher faradaic current of DA, and a differentiable oxidation peak between DA and AA in differential pulse voltammetry. Therefore, the growth surface where diamond deposition is ended is believed to be more advantageous than the nucleation side in terms of the sensitivity, selectivity, and stability of electrochemical sensing due to the high fraction of *sp*^3^ carbon.

In this paper, we present a newly developed fabrication method by which the growth surface of a BDD film can be opened up as the electrode site for chemical sensing and neural recording. The use of the exposed BDD growth side as an effective sensing surface differentiates this work from existing methods^19–21^. To demonstrate the significantly improved electrochemical properties of the growth side, we conducted a comparative study where BDD electrode probes were fabricated from either the nucleation side or the growth side of the BDD thin films produced from the same deposition batch, and their properties were compared with each other. We showed that the electrodes made from the BDD growth surface have a rougher surface, a higher *sp*^3^ fraction, a wider water potential window, and faster dynamic kinetics than the devices made from the nucleation surface. Furthermore, we validated that compared to the nucleation surface, the BDD growth surface shows better sensitivity and a lower surface absorption to DA. Finally, we assessed the biocompatibility of microcrystalline BDD films in vitro and evaluated the recording capability of the flexible BDD growth surface electrodes both in vitro using a cortical neuron culture and in vivo using the primary visual cortex (V1) of a rat.

## Results and discussion

### Fabricated device

**Fig. 1:**
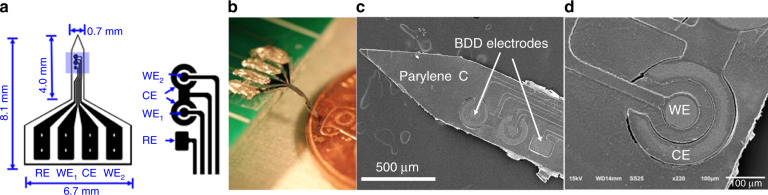
Design and fabricated prototypes of a 2-channel electrode probe for electrochemical sensing and neural recording. **a** The design of the implantable neural probe, where the areas of the WE1/WE2, RE, and CE are 0.0079, 0.028, and 0.035 mm^2^, respectively. **b**, **c** Photo and SEM images of a fabricated implantable neural probe. **d** SEM image showing the close-up view of the BDD WE and CE

Figure 1a shows the design of the proof-of-concept BDD neural probe in a three-electrode configuration. The device had two channels on a single probe shank, each of which consisted of a circular working electrode (WE) surrounded by a ring-shaped counter electrode (CE). A square reference electrode (RE) was shared by two working and counter electrode pairs. The effective areas of the exposed BDD electrodes were approximately 0.0079, 0.028, and 0.035 mm^2^ for the WE, CE, and RE, respectively. The scanning electron microscope (SEM) image in Fig. 1c shows a fabricated probe where the growth surface of the BDD film was exposed on the electrode sites. Figure 1d shows a close-up view of the WE and CE. The probe was flexible when using Parylene C as a substrate and encapsulation material, as shown in Fig. 1b.

**Fig. 2:**
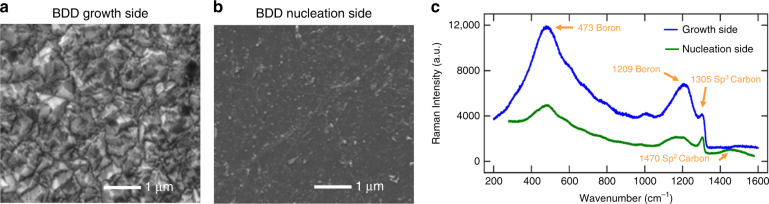
Material properties of the BDD growth side vs. the nucleation side. SEM images show the surface morphology of **a** the BDD growth side and **b** the nucleation side. **c** Raman spectrum of the BDD growth side (blue) and the nucleation side (green)

A flexible BDD probe with the nucleation side exposed was fabricated with the same geometry and compared with the BDD growth side. Corresponding SEM images and Raman spectra (532 nm Laser; HORIBA Scientific Ltd., Kyoto, Japan) of both the nucleation and growth surfaces are shown in Fig. 2a–c. The SEM images show that the growth side of the microcrystalline BDD film has a rougher surface morphology with an average grain size of 0.5 µm, while the nucleation has a relatively smooth morphology. The Raman spectra show the expected profile of the highly boron-doped diamond films in which the characteristic diamond (*sp*^*3*^) peaks at ~1332 cm^−1^ of both devices are broadened and downshifted to ~1305 cm^−1^. Additionally, two boron doping-related peaks appeared at ~473 and 1209 cm^−1^, which are the typical features of heavily doped BDD thin films with doping concentrations on the order of 10^20^ atoms/cm^3^ ^44^. This resulted in highly conductive BDD films that had a bulk resistivity in the range of 5 × 10^−3^ Ω·cm. The intensity of the boron doping-induced peak on the BDD growth side was higher than that on the BDD nucleation side since the boron dopants are distributed uniformly within the diamond grains and more boron atoms will be doped into a larger size of the diamond grains on the BDD growth side^43^. Unlike the BDD growth side, there was a small peak at ~1470 cm^−1^ for the BDD nucleation side, indicating a higher concentration of *sp*^2^ carbon and/or amorphous defects due to the smaller grain size and the larger amounts of grain boundaries. Since these non-diamond impurities are distributed along the grain boundary, the Raman intensity at 1470 cm^−1^ of the BDD nucleation side was much higher than that of the BDD growth side.

### Electrochemical properties

**Fig. 3:**
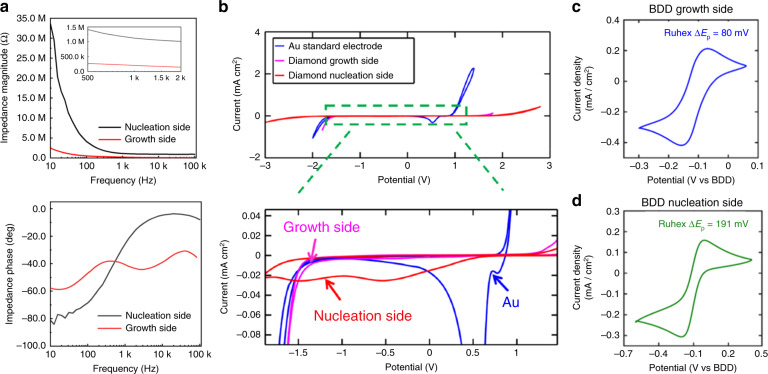
Electrochemical properties of the BDD growth surface vs. the nucleation surface. **a** Bode plots showing broadband impedances (10 Hz to 100 kHz) of the BDD growth side and nucleation side electrodes, measured in a pH 7.4 PBS buffer solution. **b** Cyclic voltammograms (CVs) of the Au and BDD growth side and nucleation side electrodes in a 1 M KCl solution (WE: BDD or Au, CE: Pt, RE: Ag/AgCl). The scan rate is 0.1 V/s. Voltammograms of the BDD growth side (**c**) and nucleation side (**d**) electrodes in the mixture of 1.5 mM Ru(NH_3_)_6_^2+/3+^ and 1.0 M KCl, scanned at a constant rate of 0.1 V/s. In this case, both the CE and RE are made of BDD

The electrochemical impedance of the BDD electrodes was measured using electrochemical impedance spectroscopy (EIS) in 0.1 M, pH = 7.4 phosphate-buffered saline (PBS) solution at room temperature. Figure 3a shows the broad impedances of the BDD electrodes with the growth side or nucleation side as the sensing surface. The 1 kHz impedance of the BDD growth side electrode is ~207.9 kΩ, which is 4–5 times lower than that of the nucleation side electrode (~1123.8 kΩ). Without changing the size of the electrodes, the BDD growth side has a rougher surface and larger grain size, which can dramatically reduce the impedance, thereby reducing the impedance noise at the electrode–electrolyte interface for neural recording. A comparative study of the potential window and background current among the diamond nucleation side, the diamond growth side, and a commercially available gold electrode was performed using cyclic voltammetry (CV) in 1.0 M potassium chloride (KCl) solution with a platinum (Pt) electrode as the CE and a silver/silver chloride (Ag/AgCl) electrode as the RE at a scan rate of 0.1 V/s. As shown in Fig. 3b, the BDD electrodes exhibited a featureless background current and wider water potential windows compared to the standard gold electrode. This characteristic permits the detection of chemical analytes in an expanded potential range of operation with reduced interference from the non-faradaic background current of the electrolyte. The background peaks presented in the nucleation side CV might be attributed to the reduction in functional groups at the *sp*^2^ sites^45^.

The effect of the electrochemical kinetics was studied using the one-electron transfer, outer-sphere electron, Ru(NH_3_)_6_^2+/3+^ redox couple. Figure 3c, d shows the cyclic voltammograms (CVs) of the BDD growth side and nucleation side electrodes, respectively, obtained in a mixture of 1.5 mM Ru(NH_3_)_6_^2+/3+^ and 1.0 M KCl at a scan rate of 0.1 V/s. The separation between the anodic and cathodic peak Δ*E*_p_ has been defined to determine the electrochemical reversibility for a given redox couple. For the one-electron transfer redox couple at room temperature, Δ*E*_p_ = 58.5 mV, which is the criterion for a complete thermodynamically reversible process^46^. The Δ*E*_p_ of the BDD growth side was 80 mV, which is close to the theoretical value of 58.5 mV, thereby indicating the quasireversibility of the reaction. The Δ*E*_p_ of BDD nucleation was 191 mV, indicating that the BDD nucleation side has much slower thermal dynamic kinetics than the BDD growth side. Background CV measurements were repeated at various scan rates of 0.1, 0.5, 1.0, 2.0, and 3.0 V/s in 1.0 M KCl (Fig. S1(a)) to assess the double-layer capacitance (*C*_dl_) of the BDD electrodes. The average current of the forward and reverse sweeps extrapolated from the CVs at different scan rates was used to derive a linear regression curve (Fig. S1(b)), where the slope defines the double-layer capacitance^42^. Using this method, the double-layer capacitance of the BDD growth side was approximately 10 µF/cm^2^, significantly lower than the *C*_dl_ of the nucleation side reported in our previous work^42^, which was ~24 µF/cm^2^. This low capacitance may result from the existence of a space charge region within the near surface of diamond electrodes^47^. The low double-layer capacitance exhibited by the BDD electrodes permits a reduced charging current and low electrochemical background noise, which has been proven to facilitate chemical sensing of electroactive species (i.e., DA) with sensitivity at least one order of magnitude greater than that of glassy carbon electrodes^48^.

### DA sensing capability

**Fig. 4:**
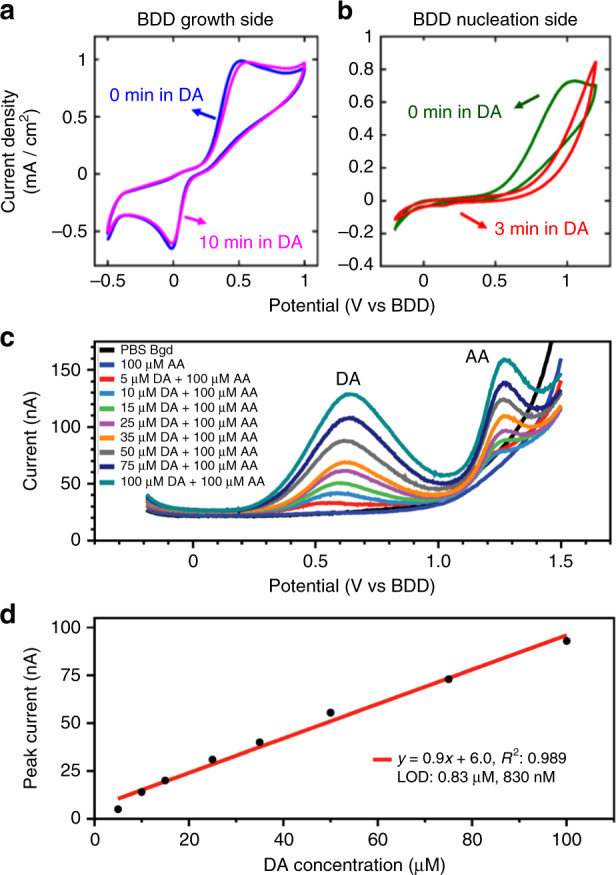
Validation of the DA sensing capability of the flexible BDD electrode. CVs of the BDD growth side (**a**) and nucleation side (**b**) electrodes in the 1.0 mM DA solution at a scan rate of 1.0 V/s. The voltammograms of both devices after extended soaking in the DA solution were also plotted as a surface fouling test. **c** SWVs of DA detection in the presence of 100 µM AA. Measurements were performed using a BDD growth side electrode. **d** Linear fitting of the peak current of SWVs under various DA concentrations of 5–100 µM

The capability of sensing the inner sphere and two-electron transfer DA/DAquinone redox couple was compared between the two surfaces. The voltammograms of the BDD growth side and the nucleation side in 1.0 mM DA diluted with a 0.1 M, pH = 7.4, phosphate-buffered saline (PBS) buffer solution at a scan rate of 1.0 V/s are shown in Fig. 4a, b, respectively. The voltammogram of the BDD growth side electrode (Fig. 4a) shows well-defined, nearly symmetrical, sharp oxidation and reduction peaks. The BDD nucleation side device (Fig. 4b), on the other hand, shows a broad oxidation peak and no significant reduction peak in the voltammogram. This result indicates that the nucleation side has a sluggish electron transfer process due to the non-diamond *sp*^*2*^ carbon. A preliminary fouling test was conducted, where both devices were soaked in a 1.0 mM DA solution to study the chemical absorption of different BDD surfaces. The DA concentration used in the experiment is much higher than the expected in vivo concentration in the brain environment^49^ to accelerate the fouling effect. Due to the non-diamond *sp*^*2*^ carbon impurity, the surface of the BDD nucleation side device was fouled within 3 min of DA soaking, resulting in the disappearance of the oxidation peak in the voltammogram, as shown in Fig. 4b. The BDD growth side device shows similar voltammograms before and after the 10-min soaking test, suggesting no significant DA absorption or fouling.

Having validated their better DA sensing performance, we evaluated the selectivity of the BDD growth side electrodes for DA detection in the presence of AA, a major biological interference for in vivo dopamine sensing^31^. In this experiment, DA at various concentrations from 5 to 100 µM was serially diluted in a mixture of 100 µM ascorbic acid and 0.1 M PBS, and these solutions were used for the square-wave voltammetry (SWV) measurements. The clear peak separation of DA from AA and from each other at different concentrations was observed in the square-wave voltammograms (SWVs) in Fig. 4c, demonstrating the selectivity of the pristine BDD growth side for DA sensing. Furthermore, the BDD growth side electrode exhibits a highly linear response with the correlation coefficient *R*^2^ = 0.989 over the tested DA concentration range, as shown in Fig. 4d. A limit of detection (LOD) of 830 nM was estimated based on linear fitting.

### In vitro assessment of diamond biocompatibility

**Fig. 5:**
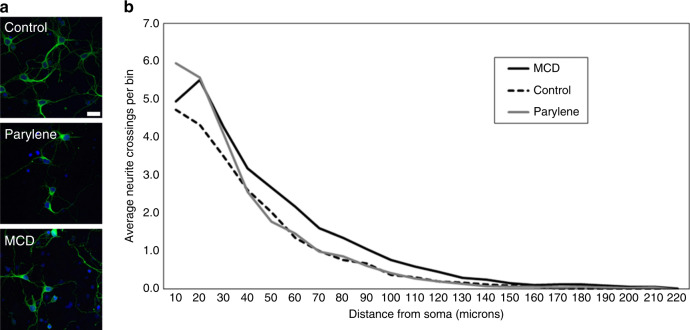
Morphological response to the electrode materials. **a** Rat cortical neurons plated on the Parylene C and MCD substrates extended neurites and displayed morphologies that appeared similar to cells plated on control substrates (TUJ1 in green, Hoechst nuclear counterstain in blue). **b** Quantification of the morphological effects via Sholl analysis illustrates similar responses on all substrates, with a slight increase in neurite extension over longer distances (>40 microns) registered by MCD substrates (*n* = ~60 neurons/substrate). Scale in (**a**) = 20 microns

Neurons seeded on microcrystalline diamond (MCD) and Parylene C appeared qualitatively similar to the control substrate in terms of the adhesion, health, and neurite outgrowth (Fig. 5a). To assess the more nuanced effects on the morphology, a Sholl analysis was performed on ~60 cells per substrate. The results revealed that the number of neurites that crossed the boundary of the concentric circles (positioned at incrementally increasing 10 µm distances from the center of each soma) was significantly higher on MCD than on Parylene C and the control substrates (Fig. 5b, linear mixed models test: *p* < 0.05). Notably, the first 20 microns displayed a slight upward trend in neurite crossings that was not evident under the other conditions tested; this may be explained by the biological variability. The “maximal distance” measurement likewise indicated that neurons plated on MCD extended longer neurites than cells cultured on the control and Parylene substrates (data not shown; linear mixed models test: *p* < 0.05). The average “number of branching” was statistically higher for Parylene and MCD than for the control, where MCD facilitated the most robust arborization (data not shown; linear mixed models test: *p* < 0.001). Last, the average sum of crossings and the average sum of branchings showed no statistical significance between the substrates (data not shown; linear mixed models test: *p* > 0.05). Additionally, positive caspase-3 staining was not detected in any of the substrates tested, indicating a similar support of the viability (the antibody was validated in preliminary tests, data not shown). While the underlying mechanism has yet to be determined, it is possible that the topographical cues presented by the granular MCD surface may promote neuronal maturation and neurite elongation^50,51^. However, the underlying mechanism has yet to be determined in future studies, and it is possible that the surface chemistry, material stiffness, and/or conductivity also play a role in this result^52,53^. Overall, the MCD and Parylene C substrates performed as expected in vitro and supported neuronal growth and maturation similarly to the control conditions.

### In vitro extracellular recording

**Fig. 6:**
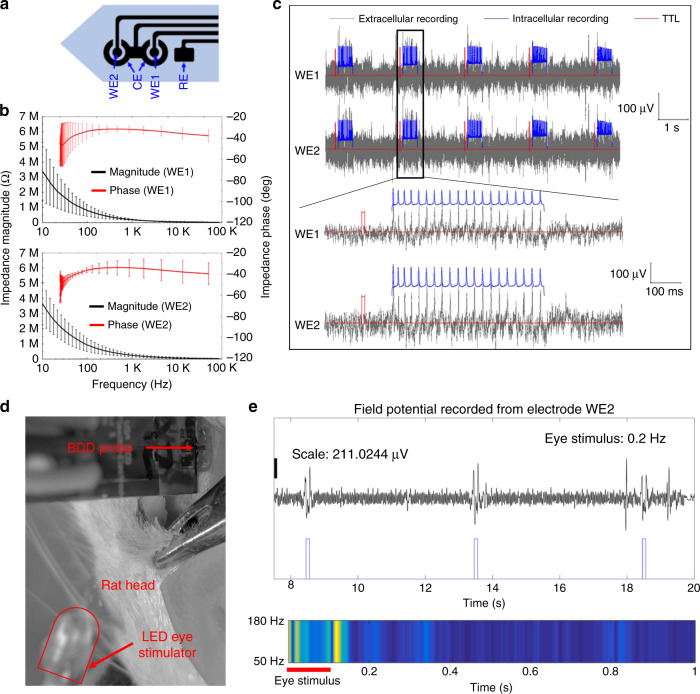
In vitro and in vivo extracellular recordings using flexible BDD growth surface electrodes. **a** Electrode layout. **b** Bode plots showing impedances (10 Hz to 100 kHz) of the WE1 and WE2 BDD electrodes measured in the pH 7.4 PBS buffer solution (sample size: *n* = 4). **c** Extracellular recording from the cultured cortical neurons, showing that the spikes detected by the BDD electrode are time-locked to the intracellular activity registered by the patch electrode. **d** Setup of in vivo neural recording in the V1 of a male rat. **e** Visually induced field potential recorded through the BDD electrode WE2 (top). The color chart (bottom) shows the spectrogram (50–180 Hz) of the recording within the 1-s cycle. The square wave is the pulses of the eye stimulus

The electrochemical impedance of the WEs was measured using EIS in a room temperature PBS solution (0.1 M, pH = 7.4). Figure 6b shows that the average 1 kHz impedance (*n* = 4) of the WE1 and WE2 is approximately 254.2 and 203.4 kΩ, respectively, which are suitable for neurophysiology recording. The intracellular spiking activity of cultured neurons detected with patch-clamp electrophysiology was highly coincident with the extracellular activity recorded from the two-channel flexible BDD electrode probes (Fig. 6c), demonstrating that the flexible electrodes are capable of recording the extracellular activity of proximal neurons. Previous studies have reported simultaneous intra/extracellular “ground-truth” recordings using comparable configurations, such as glass pipette electrodes inserted near implanted tetrodes in vivo^54,55^ or through the use of microelectrode arrays as neuronal culture substrates in vitro^56,57^. Based on visual estimates of the peak-to-peak noise floor, the signal-to-noise ratio (SNR) of the flexible BDD devices appeared somewhat lower (SNR ~ 2) than traces reported in alternative setups (SNR ~ 3–4 in ref. ^54^). While the purpose of the in vitro recording was to provide proof-of-principle for subsequent in vivo use, the SNR of Parylene-BDD electrodes may be further improved in future studies by reducing external noise or optimizing device design (e.g., increasing the surface area of recording sites to reduce impedance and thermal noise).

### In vivo electrophysiology recording

Figure 6d shows the setup of in vivo experiments using the rat model. The probe was inserted in the left lobe of the V1 region at a penetration depth of approximately 1 mm. During the experiments, the right eye of the rat was stimulated optically using a blue LED at a 100 ms pulse width and a 0.2 Hz frequency. The BDD electrode that is the closest to the probe tip (WE2) was used to record spontaneous neural responses from the left V1. Figure 6e shows the visually induced local field potential recorded through WE2, where elevated field potentials were detected shortly after visual stimulation, demonstrating stimulus-induced neural activity. The recorded field potential signal was truncated to a 1-s trial (total of 240 trials), averaged across 240 trials, and mapped onto a color-coded time-varying energy distribution (spectrogram) in the gamma band (50–180 Hz) within the 1-s time window. As shown in Fig. 6e (bottom), higher gamma band energy was observed within a 50 ms window post-stimulation, which is consistent with our previous observation^58^. While preliminary, this result suggests that the BDD electrodes are capable of recording neural activity from living tissues.

## Methods and materials

### Device fabrication

**Fig. 7:**
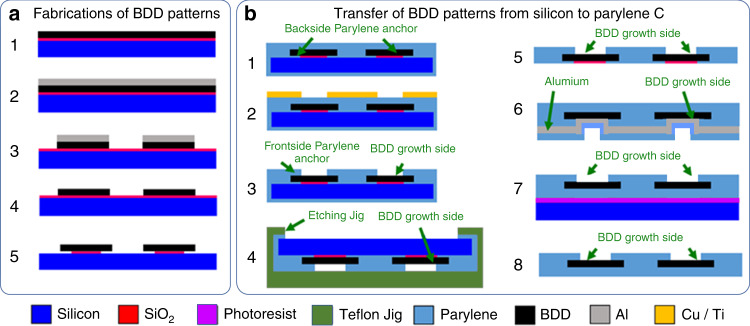
Illustration of the fabrication process flow. **a** Growth and patterning of the BDD structures and **b** approach to transfer the prepatterned BDD structures from a silicon wafer onto a Parylene C thin film substrate

Figure 7a illustrates the fabrication process flow for BDD synthesis and patterning. Four-inch-diameter silicon wafers were cleaned followed by chemical vapor deposition of 1 µm silicon dioxide. Then, the wafers were scratch seeded, and microcrystalline BDD films were grown on the wafers using a 915 MHz microwave plasma-assisted chemical vapor deposition (MW-PACVD) reactor dedicated for high-quality born-doped diamond thin film growth. The standard BDD synthesis conditions were as follows: a microwave power of 8 kW, a stage temperature of 850 °C, a chamber pressure of 65 Torr, and a gas chemistry of 1% methane (CH_4_) in a hydrogen (H_2_) balance. Diborane (B_2_H_6_) was added to the diamond growth process in a B/C ratio of 37,500 ppm to achieve sufficiently high conductivity. After the BDD synthesis, aluminum was thermally evaporated (Auto 306; Edward, Inc., West Sussex, UK) and patterned via ultraviolet (UV) photolithography (ABM-USA, Inc., San Jose, CA, USA), followed by wet chemical etching in an aluminum etchant (Type A; Transene Co., Inc., Danvers, MA, USA). With the patterned aluminum mask, the BDD film was plasma etched in an electron cyclotron resonance reactive ion etcher (RIE) using SF_6_/Ar/O_2_ with a microwave power of 1000 W and a radio-frequency (RF) bias of 150 W (180 V). Afterwards, the aluminum mask was removed using the aluminum etchant before the SiO_2_ was etched in a buffered oxide etchant (BOE). The SiO_2_ layer was slightly overetched undercutting the BDD structures to form Parylene anchors for the subsequent transfer processes.

Figure 7b depicts the transfer process where the prepatterned BDD structures were transferred onto Parylene C films with the growth side exposed as the sensing electrodes. For this purpose, ~15 μm Parylene C was chemically vapor deposited on the patterned BDD film at room temperature (PDS 2010, Specialty Coating System, Inc., Indianapolis, IN, USA). Prior to Parylene deposition, the wafer was treated with a Silane A174 adhesion promoter (Sigma Aldrich, Inc., St. Louis, MO, USA). Titanium/copper was thermally evaporated and patterned to form a mask for the plasma etching of Parylene C. Parylene was etched utilizing an oxygen RF plasma with an overall power of 300 W and gas flow rate of ~50 sccm (PX-250; Nordson March, Inc., Concord, CA, USA). This mild plasma etch was chosen to minimize the damage to the BDD surface since oxygen plasma also etches diamond. Preparing for silicon removal, the Parylene C coating on the backside of the silicon wafer was removed with the oxygen plasma using the above recipe. The wafer was then placed upside down into a custom-made jig and sealed with an O-ring. The exposed silicon substrate was completely etched in 35% KOH at 70 °C to release the BDD-Parylene C film. After removing the residual SiO_2_ from the nucleation side, aluminum was deposited on the growth side, followed by the deposition of another 10 μm Parylene C layer to encapsulate the exposed nucleation side. The BDD-Parylene film was then glued on a silicon carrier wafer using photoresist, with the growth side facing upwards. The Parylene C on top of aluminum was plasma etched, followed by aluminum removal using an aluminum etchant to expose the BDD growth side as the recording electrodes. Finally, the BDD-Parylene film was released from the carrier substrate by dissolving the sacrificial photoresist in acetone and rinsed with isopropyl alcohol and deionized water. To make a fair comparison, microelectrode probes where the BDD nucleation side was exposed were fabricated using BDD films from the same MW-PACVD batch based on our previously developed method^42^.

### Material characterization

The surface morphology of the BDD nucleation and growth sides was studied using SEM (6610 V; JEOL Inc., Peabody, MA, USA). The surface chemistry of both sides was analyzed using Raman spectroscopy (532 nm Laser; HORIBA Scientific Inc., Kyoto, Japan). The surface resistivity of the BDD film was measured using a four-point probe. The electrochemical impedance of the electrodes was measured using EIS (CHI604; CH Instruments, Inc., Austin, TX, USA). The electrochemical properties of the BDD electrodes were characterized using CV (CHI604; CH Instruments, Inc., Austin, TX, USA) in a three-electrode configuration. During the experiments, the DA solution was prepared by dissolving DA in a 0.1 M, pH = 7.4 PBS buffer solution. The effect of AA interference on DA detection was evaluated using SWV measurements (CHI604; CH Instruments, Inc., Austin, TX, USA). Experimental solutions were prepared by serially diluting DA in a mixture of 100 µM AA and 0.1 M, pH = 7.4 PBS. For the three-electrode setup, an Ag/AgCl reference electrode (CHI 111; CH Instruments, Inc., Austin, TX, USA) was used, while a commercially available Pt wire (CHI 102; CH Instruments, Inc., Austin, TX, USA) was used as the counter electrode. Hexaammine-ruthenium (III) chloride (Ru(NH_3_)_6_Cl), KCl, DA, and AA were purchased from Sigma Aldrich.

### In vitro biocompatibility assessment

To assess the growth and attachment of neurons on the Parylene and diamond materials used in the device, E18 embryonic rat cortical neurons (#A1084002; Thermo Fisher Scientific, Waltham, MA, USA) were cultured on the surface of planar substrates fabricated from these materials following poly-d-lysine coating according to the supplier’s protocol (#A3890401; Thermo Fisher Scientific, Waltham, MA, USA). Neurons plated on poly-d-lysine-coated Nunc Thermanox plastic coverslips (#174950; Thermo Fisher Scientific, Waltham, MA, USA) were used as a control. Neurons were fixed with 4% paraformaldehyde in PBS at the 7-day time point and stained for beta III-tubulin (TUJ1) and caspase-3 to assess the neurite outgrowth and viability, respectively. Immunohistochemistry was performed using previously reported methods^52^; the primary antibodies used were a monoclonal mouse anti-beta III-tubulin antibody (“TUJ1”, #ab78078; Abcam, Cambridge, UK) at a 1:500 dilution and a rabbit cleaved caspase-3 antibody (#9661S; Cell Signaling Technology, Inc., Danvers, MA, USA) at a 1:400 dilution. Similarly, the secondary antibodies used were Alexa Fluor goat anti-rabbit 594 (#A-11037; Thermo Fisher Scientific, Waltham, MA, USA) at a 1:200 dilution and Alexa Fluor anti-mouse 488 (#A-11001; Thermo Fisher Scientific, Waltham, MA, USA) at a 1:200 dilution. Sholl analysis was performed to assess the neurite outgrowth according to published protocols^59^, based on previous studies that demonstrate its utility as a measure of biocompatibility^60^. Briefly, concentric circles with defined radii of 10 µm were centered on the neuronal soma and used to manually benchmark the length, branching, and intersection of the neurite outgrowth. The “maximal distance” is defined as a measurement of the maximum distance that any individual neurite extends from the soma. The “number of branching” is defined as a measurement of how many branches are formed in each particular bin. An average of ~60 neurons for each condition was randomly selected and analyzed if the neurites were clearly identifiable.

### In vitro electrophysiology recording

For these experiments, rat cortical neurons were cultured on plastic coverslips, and whole-cell patch-clamp electrophysiology was performed using previously reported methods^52^. While the patch electrode accessed the neuronal membrane, a flexible BDD probe was carefully positioned adjacent to the recorded neuron in the solution using a separate micromanipulator. The patch digitizer was programmed to deliver a 5 V TTL pulse to an Intan RHA 2000 board 100 ms prior to the delivery of a 500 ms epoch of intracellular stimulation. At the same time, the extracellular activity was recorded via the BDD electrodes using an Intan RHA2216 amplifier chip and RHA 2000 evaluation board (Intan Technologies LLC, Los Angeles, CA, USA).

### In vivo electrophysiology recording

To validate the device functionality for in vivo neural recording, the flexible diamond probe was implanted in the V1 of an adult Sprague Dawley rat (male, ~550 g) using stereotaxic surgery procedures that were approved by the Institutional Animal Care and Use Committee (IACUC) at Michigan State University. During the surgery, the rat was fixed on a stereotaxic frame and anesthetized with a 2–4% isoflurane and oxygen mixture. A 3–4 cm incision was made in the skin overlying the skull, and a unilateral craniotomy was made on the left V1 (lateral: 3.6 mm, anteroposterior: 6.3 mm relative to Bregma) using a precision surgical drill. To remove the dura mater, the bevel of a 30-gauge sterile syringe needle was pressed against a solid surface to form a small hook. The dura mater was carefully pierced and lifted using the hook and then cut using microscissors^61^ to expose the cortical tissue. For device implantation, the flexible probe was coated with polyethylene glycol (PEG) to temporarily stiffen the device during insertion into the cortex. The PEG-coated probe was placed in a micromanipulator mounted on the stereotaxic frame and manually lowered to the desired stereotaxic location of the brain. During the insertion, sterile saline was applied on the surface of the brain to dissolve the PEG coating from a small portion of the implant above the brain surface as the probe was advanced to the desired depth of the V1^62^. For neural recording, the BDD electrodes were connected to a commercial 32-channel Intan recording chip, and the recorded signals were amplified and digitalized by an Intan RHD2132 system (Intan Technologies, Los Angeles, CA, USA). A ground electrode was attached to the tissue around the neck area. In some recording trials, the right eye of the rat was stimulated with LED light pulses, and the left eye was covered to avoid direct exposure to the light stimulus.

## Conclusion

In this paper, we designed, fabricated, and tested a flexible BDD neural probe, where the BDD growth surface was utilized as the electrode site for neurophysiology and neurochemical sensing with potentially improved sensitivity and stability. A fabrication method was developed to transfer the BDD patterns from the solid silicon substrate onto the flexible polymer substrate and to expose the BDD growth side as the sensing surface. The general material properties and electrochemical characteristics were compared between the BDD nucleation and growth surfaces. Our results demonstrated that the electrodes made of the BDD growth side exhibited a rougher topology, a higher *sp*^3^ content, and a larger grain size than the nucleation side. All of these results are attributed to the better sensitivity and stability of the BDD growth side electrodes for DA sensing and neural recording. In particular, for electrochemical sensing, a high-quality diamond surface with *sp*^*3*^ carbon enables a wide working potential window, low background noise (due to the low double-layer capacitance), and a resistance to chemical fouling. Consequently, electrodes made of the BDD growth surface are expected to provide a wide polarizable range of chemical reactions in electrochemical sensing with improved sensitivity and reliability. On the other hand, the nanoscale surface roughness and large grain size increase the effective surface area of the electrode, therefore reducing the electrochemical impedance for electrophysiology recording with reduced impedance noise. The neural recording capability of the BDD growth side electrodes was validated both in vitro and in vivo. This flexible BDD microelectrode technology is expected to provide a unique tool for simultaneous neurophysiology and neurotransmitter sensing in neuronal circuits, which can open up numerous opportunities for fundamental and clinical research on a wide variety of brain disorders and diseases, such as Parkinson’s disease.

## Supplementary information

Editorial summary
