# Supplementary material for: Flexible, diamond-based microelectrodes fabricated using the diamond growth side for neural sensing
Source: Microsyst Nanoeng. 2020 Jul 13;6:42. doi: 10.1038/s41378-020-0155-1 (PMC7355183; doi:10.1038/s41378-020-0155-1)
Supplement: Supplementary file 1 — Editorial summary [file 41378_2020_155_MOESM1_ESM.docx]

**Flexible, Diamond-based Microelectrodes Fabricated Using the Diamond Growth Side for Neural Sensing**

*Bin Fan^1^, Cory A. Rusinek^2^, Cort H. Thompson^3^, Monica Setien^3^, Yue Guo^1^, Robert Rechenberg^2^, Yan Gong^1^, Arthur J. Weber^4^, Michael F. Becker^2^, Erin Purcell^3,1^, Wen Li^1^,*

^1^Department of Electrical and Computer Engineering, Michigan State University, East Lansing, Michigan, USA

^2^Fraunhofer USA, Center for Coatings and Diamond Technologies, East Lansing, Michigan, USA

^3^Department of Biomedical Engineering, Michigan State University, East Lansing, Michigan, USA

^4^Department of Physiology, Michigan State University, East Lansing, Michigan, USA

Correspondence: Wen Li (wenli@msu.edu)

^1^Department of Electrical and Computer Engineering, Michigan State University, East Lansing, USA.

Supplemental Materials

1. Double-layer capacitance

To quantify the double-layer capacitance (C_dl_) of the BDD electrode experimentally, cyclic voltammetry (CV) measurements were performed in 1.0 M KCl solution with various scan rates. During the measurements, all the three electrodes on the same BDD probe were used as RE, CE, and WE. To give an example, Fig. S1(a) shows the cyclic voltammograms (CVs) of a BDD nucleation side electrode at scan rates of 0.1, 0.5, 1.0, 2.0 and 3.0 V/s. A linear regression curve (Fig. S1(b)) was derived from the average current of forward and reverse sweeps at −0.9 V vs. BDD with an R^2^ number of 0.979, where the slope indicates the C_dl_ of 24 μF/cm^2^.


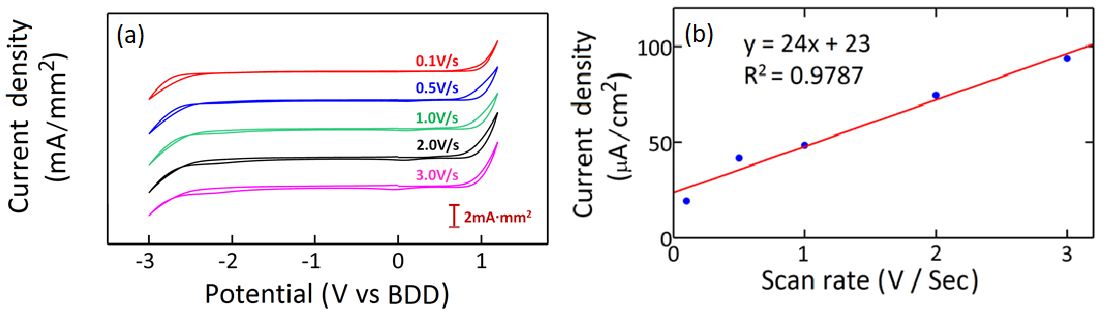


Fig. S1: Double-layer capacitance extrapolated from CVs at varied scan rates. (a) Voltammograms of BDD electrodes vs. BDD at various scan rates (CE: BDD, RE: BDD). The voltammograms are offset for better visibility. (b) The linear regression curve of the average current at different scan rates.
